# Identification by PCR of Non-typhoidal *Salmonella enterica* Serovars Associated with Invasive Infections among Febrile Patients in Mali

**DOI:** 10.1371/journal.pntd.0000621

**Published:** 2010-03-09

**Authors:** Sharon M. Tennant, Souleymane Diallo, Haim Levy, Sofie Livio, Samba O. Sow, Milagritos Tapia, Patricia I. Fields, Matthew Mikoleit, Boubou Tamboura, Karen L. Kotloff, James P. Nataro, James E. Galen, Myron M. Levine

**Affiliations:** 1 Center for Vaccine Development, University of Maryland School of Medicine, Baltimore, Maryland, United States of America; 2 Centre pour le Développement des Vaccins (CVD-Mali), Bamako, Mali; 3 Hôpital Gabriel Touré, Bamako, Mali; 4 Israel Institute for Biological Research, Ness Ziona, Israel; 5 National Salmonella Reference Laboratory, Centers for Disease Control and Prevention, Atlanta, Georgia, United States of America; Cambridge University, United Kingdom

## Abstract

**Background:**

In sub-Saharan Africa, non-typhoidal *Salmonella* (NTS) are emerging as a prominent cause of invasive disease (bacteremia and focal infections such as meningitis) in infants and young children. Importantly, including data from Mali, three serovars, *Salmonella enterica* serovar Typhimurium, *Salmonella* Enteritidis and *Salmonella* Dublin, account for the majority of non-typhoidal *Salmonella* isolated from these patients.

**Methods:**

We have extended a previously developed series of polymerase chain reactions (PCRs) based on O serogrouping and H typing to identify *Salmonella* Typhimurium and variants (mostly I 4,[5],12:i:-), *Salmonella* Enteritidis and *Salmonella* Dublin. We also designed primers to detect *Salmonella* Stanleyville, a serovar found in West Africa. Another PCR was used to differentiate diphasic *Salmonella* Typhimurium and monophasic *Salmonella* Typhimurium from other O serogroup B, H:i serovars. We used these PCRs to blind-test 327 *Salmonella* serogroup B and D isolates that were obtained from the blood cultures of febrile patients in Bamako, Mali.

**Principal Findings:**

We have shown that when used in conjunction with our previously described O-serogrouping PCR, our PCRs are 100% sensitive and specific in identifying *Salmonella* Typhimurium and variants, *Salmonella* Enteritidis, *Salmonella* Dublin and *Salmonella* Stanleyville. When we attempted to differentiate 171 *Salmonella* Typhimurium (I 4,[ 5],12:i:1,2) strains from 52 monophasic *Salmonella* Typhimurium (I 4,[5],12:i:-) strains, we were able to correctly identify 170 of the *Salmonella* Typhimurium and 51 of the *Salmonella* I 4,[5],12:i:- strains.

**Conclusion:**

We have described a simple yet effective PCR method to support surveillance of the incidence of invasive disease caused by NTS in developing countries.

## Author Summary

The genus *Salmonella* has more than 2500 serological variants (serovars), such as *Salmonella enterica* serovar Typhi and *Salmonella* Paratyphi A and B, that cause, respectively, typhoid and paratyphoid fevers (enteric fevers), and a large number of non-typhoidal *Salmonella* (NTS) serovars that cause gastroenteritis in healthy hosts. In young infants, the elderly and immunocompromised hosts, NTS can cause severe, fatal invasive disease. Multiple studies of pediatric patients in sub-Saharan Africa have documented the important role of NTS, in particular *Salmonella* Typhimurium and *Salmonella* Enteritidis (and to a lesser degree *Salmonella* Dublin), as invasive bacterial pathogens. *Salmonella* spp. are isolated from blood and identified by standard microbiological techniques and the serovar is ascertained by agglutination with commercial antisera. PCR-based typing techniques are becoming increasingly popular in developing countries, in part because high quality typing sera are difficult to obtain and expensive and H serotyping is technically difficult. We have developed a series of polymerase chain reactions (PCRs) to identify *Salmonella* Typhimurium and variants, *Salmonella* Enteritidis and *Salmonella* Dublin. We successfully identified 327 *Salmonella* isolates using our multiplex PCR. We also designed primers to detect *Salmonella* Stanleyville, a serovar found in West Africa. Another PCR generally differentiated diphasic *Salmonella* Typhimurium and monophasic *Salmonella* Typhimurium variant strains from other closely related strains. The PCRs described here will enable more laboratories in developing countries to serotype NTS that have been isolated from blood.

## Introduction

In industrialized countries, non-typhoidal *Salmonella* (NTS) constitute a well recognized public health problem that in healthy subjects is overwhelmingly encountered clinically as self-limited gastroenteritis [1],[2]. In immunocompromised and debilitated hosts, NTS can become invasive, leading to bacteremia, sepsis and focal infections (e.g., meningitis) [2],[3]. Among infants less than three months of age who become infected with NTS in industrialized countries, invasiveness is also occasionally observed, resulting in bacteremia and focal infections [4].

Interestingly, whereas systematic blood culture-based surveillance of febrile pediatric patients in Asia has clearly highlighted the high incidence of bacteremia associated with *Salmonella enterica* serovars Typhi and Paratyphi A in children residing in crowded urban settings [5]–[7], isolation of NTS has not been common. In striking contrast, systematic blood culture-based surveillance and clinical studies of hospitalized and ambulatory pediatric patients <60 months of age with fever or focal infections in sub-Saharan Africa have documented the important role of NTS as invasive bacterial pathogens [8]–[17]. NTS constituted one of the three most common invasive bacterial pathogens in all these studies. Importantly, two serovars, *Salmonella* Typhimurium (and Typhimurium variants) and *Salmonella* Enteritidis have been reported to account for 79–95% of all bacteremic non-typhoidal *Salmonella* infections in sub-Saharan Africa [9], [11]–[13],[15],[16],[18],[19]. *Salmonella* Dublin has been associated with a few percent of cases in some studies [12],[13] but with a more substantial proportion in Mali [18], where a fourth serovar, *Salmonella* Stanleyville, also accounted for a notable proportion of all isolates [18], bringing the cumulative total to >95% of all strains.

We previously developed a multiplex polymerase chain reaction (PCR)-based approach to identify the three main pathogens responsible for typhoid (*Salmonella* Typhi) and paratyphoid (*Salmonella* Paratyphi A and *Salmonella* Paratyphi B) fevers [18]. Three sequential PCRs identify strains of *Salmonella* serogroups A, B or D (and Vi positive or negative); strains that express Phase 1 flagellar (H) antigen types H:a, H:b or H:d; and strains incapable of fermenting d-tartrate (d-T). By means of this PCR technology, *Salmonella* Typhi (O serogroup D, Vi^+^; H:d), *Salmonella* Paratyphi A (O serogroup A; H:a) and *Salmonella* Paratyphi B (O serogroup B; H:b; d-T non-fermenter) strains were identified with 100% sensitivity and 100% specificity.

Classical *Salmonella* serotyping methods identified the serovars of 336 NTS isolates from blood cultures of febrile children <16 years of age in Bamako, Mali, obtained in the course of systematic surveillance of children admitted to hospital or seen in the Emergency Department with fever or invasive infection syndromes [20]–[22]. *Salmonella* Typhimurium and “variants” (mainly I 4,[5],12:i:-), *Salmonella* Dublin, *Salmonella* Enteritidis and *Salmonella* Stanleyville were the most commonly isolated NTS [18]. Herein, we describe PCRs that when used in conjunction with the O serogrouping PCR described by Levy et al. [18] can identify *Salmonella* Typhimurium and variants (O serogroup B; H:i), *Salmonella* Enteritidis (O serogroup D, Vi^-^; H:g,m), *Salmonella* Dublin (O serogroup D, Vi^+^ or Vi^-^; H:g,p) and *Salmonella* Stanleyville (O serogroup B; H:z4,z23) with 100% sensitivity and 100% specificity. We anticipate that this methodology will be useful in reference laboratories and major clinical microbiology laboratories to identify *Salmonella* isolated from blood and other sterile sites in developing countries where robust PCR-based typing techniques are becoming increasingly popular and because high quality H typing sera are difficult to obtain, expensive and technically demanding to use.

## Methods

### Ethics statement

The surveillance protocol and consent form were reviewed by the Ethics Committee of the Faculté de Médecine, Pharmacie et Odonto-Stomatologie, Université de Bamako, and by the Institutional Review Board of the University of Maryland, Baltimore. For any patient eligible for laboratory surveillance to detect invasive bacterial disease, informed consent was obtained prior to their enrollment; ∼95% of eligible subjects agreed to participate. Since the literacy rate in Bamako is <30%, as is customary practice for CVD-Mali clinical studies [20]–[22], the consent form was translated into Bambara and several other local languages and the translations recorded on audiotape [20]. CVD-Mali personnel explain the study, including the objectives and risks and benefits associated with participation. The audiotaped version of the consent form is then played and any questions posed are answered. Once the parent or patient has had all questions answered and agrees to participate, this is documented on a printed consent form written in French. If the participant is illiterate, a witness who is present throughout the consent procedure completes the necessary portions and signs the consent form; the parent/participant marks the consent form (either fingerprint or other notation). If the person is literate, then he/she may read and sign the consent form. This standard method of obtaining consent practiced by CVD-Mali was approved by ethics commitees in Mali and at the University of Maryland.

### Systematic surveillance for invasive bacterial infections

Since July 2002, clinical staff of the Centre pour le Développement des Vaccins du Mali (CVD-Mali) and l'Hôpital Gabriel Touré (HGT) have been conducting systematic surveillance to detect invasive bacterial disease among hospitalized children <16 years of age [20]–[22]. Age-eligible children presenting to the emergency department with fever (≥39°C) or focal clinical findings suggestive of invasive bacterial infection (meningitis, septic arthritis, etc.) and requiring hospitalization are referred to CVD-Mali staff by the evaluating clinicians. A CVD-Mali physician obtains informed consent, records clinical and epidemiologic data, and obtains blood (and other relevant fluids) for culture in the HGT Clinical Bacteriology Laboratory. The child's clinician is promptly notified when a culture yields a bacterial pathogen.

### *Salmonella* strains

*Salmonella* Typhimurium strain 81.23500, *Salmonella* Enteritidis strain CVD SE and *Salmonella* Dublin strain 06-0707 were used to develop the multiplex PCR. Twenty-four control strains which came from the *Salmonella* Reference Laboratory of the Centers for Disease Control and Prevention (CDC), Atlanta, GA or the Center for Vaccine Development, Baltimore, MD have previously been described [18]. These strains were *Salmonella* serovars of various O serogroups (B, C1, C2, D, E1, O28 and O38) and H types (b, c, d, h, i, g, k, l, m, p, s, t, v, y, z10 and z29). Nine O serogroup B, Phase 1 flagella antigen H:i reference strains from the CDC were used to develop a PCR that discriminates between *Salmonella* Typhimurium and I 4,[5],12:i:- (Table 1). The NTS test strains consist of 327 *Salmonella* serogroup B and D isolates that were originally obtained from the blood cultures of febrile patients at l'Hôpital Gabriel Touré in Bamako, Mali. These strains were identified by conventional microbiological and classical serotyping methods by the CVD and CDC, as previously described [18]; 69 isolates were O serogroup D, including 37 *Salmonella* Dublin and 32 *Salmonella* Enteritidis, and 258 isolates were O serogroup B.

**Table 1:** Nine reference strains of *Salmonella* consisting of serovars that belong to O group B and that possess Phase 1 H flagella antigen “i”.

Strain	Serovar	O antigens	Phase 2 H flagella antigen(s)
CDC 443	Gloucester	1,4,12,(27)	l,w
CDC 513	Agama	4,12	1,6
CDC 1045	Lagos	1,4,12	1,5
CDC 1638	Tsevie	4,12	e,n,z15
CDC 1855	Lagos	1,4,12	1,5
CDC 2322	Farsta	4,12	e,n,x
CDC 2419	Tumodi	1,4,12	z6
CDC 07-0794	I 4,[5],12:i:-	4,[5],12	–
CDC 07-0972	I 4,[5],12:i:-	4,[5],12	–

### Primers

#### Detection of *Salmonella* Typhimurium and variants, *Salmonella* Enteritidis and *Salmonella* Dublin (and later *Salmonella* Stanleyville)

This primer mix contained the following primers: H-for, a primer sequence common to *fliC* of both *Salmonella* Typhimurium (H:i) and *Salmonella* Dublin (H:g,p); Hi, unique to *fliC* of H:i organisms; Hgp, unique to *fliC* of H:g,p organisms; sdfF and sdfR, which amplify Sdf I, a fragment of DNA unique to *Salmonella* Enteritidis; and 16SF and DG74, universal bacterial 16S rRNA gene primers that were included to ensure that DNA was added (Table 2).

**Table 2:** Primers used in this study.

Primer	Sequence (5′ to 3′)	Amplicon (bp)	Reference
H-for	ACTCAGGCTTCCCGTAACGC		[18]
Hgp	ATTAACATCCGCCGCGCCAA	779	This study
Hi	ATAGCCATTTACCAGTTCC	551	[37]
Hz4,z23F	TTTGTCAAAGATGTTACTGCG	427	This study
Hz4,z23R	AGGTTAGTGATGGCAGATTC		This study
sdfF	TGTGTTTTATCTGATGCAAGAGG	333	[27]
sdfR	CGTTCTTCTGGTACTTACGATGAC		[27]
16SF	AATACGTTCCCGGGCCTTG	167	Based on universal bacterial probe RDR245 in [47]
DG74	AGGAGGTGATCCAACCGCA		[47]
Sense-59	CAACAACAACCTGCAGCGTGTGCG	1389	[26]
Antisense-83	GCCATATTTCAGCCTCTCGCCCG		[26]
FFLIB	CTGGCGACGATCTGTCGATG	250 or 1000	[24]
RFLIA	GCGGTATACAGTGAATTCAC		[24]

Primer Hgp was designed by performing Clustal W alignments between *fliC* nucleotide sequences deposited in GenBank (three *Salmonella* Enteritidis sequences, DQ095560, AY649709 and AY649742; one *Salmonella* Dublin sequence, AY649712). The *fliC* alleles of *Salmonella* Enteritidis (*fliC-*g,m) and *Salmonella* Dublin (*fliC-*g,p) are very similar, as they are both G complex alleles that are almost identical to one another [23]. The nucleotide sequence of *fliC-*g,p, to which primer Hgp binds, differs from *fliC-*g,m by two nucleotides. The Hgp primer was therefore designed such that one of these mismatches was at the 3′ end of Hgp (*fliC-*g,p has a ‘T’ whereas *fliC-*g,m has a ‘C’).

In a later step, primers to detect *fliC* of *Salmonella* Stanleyville (H:z4,z23) were added to the primer mix. Primers Hz4,z23F and Hz4,z23R were designed to amplify a 427-bp fragment of *fliC-*z4,z23 (based on GenBank accession no. AY649736).

#### Differentiation of diphasic *Salmonella* Typhimurium and monophasic *Salmonella* Typhimurium from other H:i serovars

We combined published primers in a PCR to discriminate between *Salmonella* Typhimurium (I 4,[5],12:i:1,2) and the monophasic serovar I 4,[5],12:i:- and other O serogroup B, H:i serovars. To accomplish this, we used a primer mix containing primers FFLIB and RFLIA, which amplify the *fliB-fliA* intergenic region, and primers Sense-59 and Antisense-83, which amplify the Phase 2 (*fljB*) flagellar gene.

### DNA extraction and PCR

PCR was performed in 1× PCR buffer, 3.5 mM MgCl_2_, 0.2 mM of dNTPs and 0.2 U of Invitrogen Taq DNA polymerase (final volume of 25 µl) in an Eppendorf Mastercycler®. The primer mixes contained primers at a concentration of 5 µM each (final concentration of 0.2 µM) except for FFLIB and RFLIA that were used at a concentration of 10 µM each and the positive control primers (16SF and DG74) that were used at a concentration of 2.5 µM each. For each PCR reaction, 1.0 µl of primer mix was used. Crude DNA was prepared by suspending 3 colonies in 100 µl water and boiling for 10 min followed by centrifugation at 16,000*×g* for 30 sec and purified DNA was prepared using a GNOME DNA kit (QBIOgene, Irvine, CA) according to the manufacturer's instructions, and 5 µl of DNA was used in each PCR. The cycling parameters of the multiplex PCR that detects H:i, H:g,p and Sdf I involved denaturation at 95°C for 2 min, followed by 25 cycles comprised of heating to 95°C for 30 sec, 64°C for 30 sec and 72°C for 15 sec, and a final step of 72°C for 5 min. The cycling parameters of the PCR that discriminates between *Salmonella* Typhimurium and I 4,[5],12:i:- involved denaturation at 95°C for 2 min, followed by 25 cycles of 95°C for 30 sec, 64°C for 30 sec and 72°C for 1.5 min, and a final step of 72°C for 5 min. PCR products were separated on 2% (w/v) agarose gels, stained with ethidium bromide and visualized using a UV transilluminator.

## Results

### A multiplex PCR to detect *Salmonella* Typhimurium and monophasic variants, *Salmonella* Enteritidis and *Salmonella* Dublin

**Figure 1:**
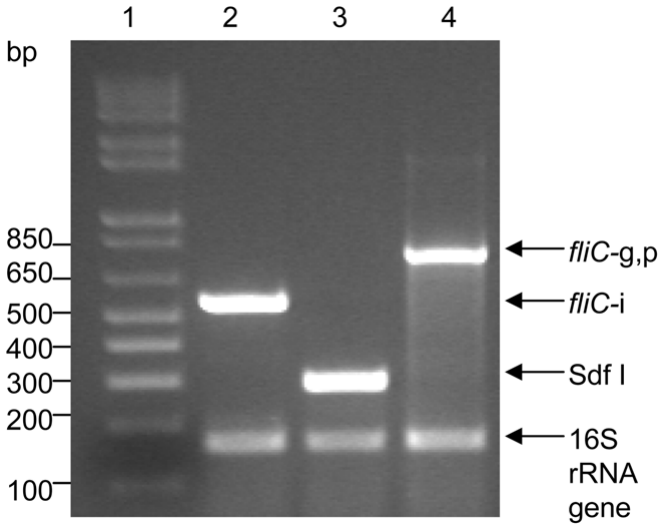
Multiplex PCR to identify *Salmonella* Typhimurium and variants, *Salmonella* Enteritidis and *Salmonella* Dublin. Lanes: 1, 1 kb ladder (Invitrogen); 2, *Salmonella* Typhimurium 81.23500; 3, *Salmonella* Enteritidis CVD SE; and 4, *Salmonella* Dublin 06-0707.

Figure 1 shows that the primers within the multiplex PCR were able to clearly identify the appropriate NTS alleles. A 779-bp product was amplified from *Salmonella* Dublin (*fliC*-gp), a 551-bp product was amplified from *Salmonella* Typhimurium (*fliC*-i) and a 333-bp product was amplified from *Salmonella* Enteritidis (Sdf I). The internal positive control primers (universal 16S rRNA gene primers) amplified a 167-bp product from each strain.

### Initial validation of the multiplex PCR assay

**Figure 2:**
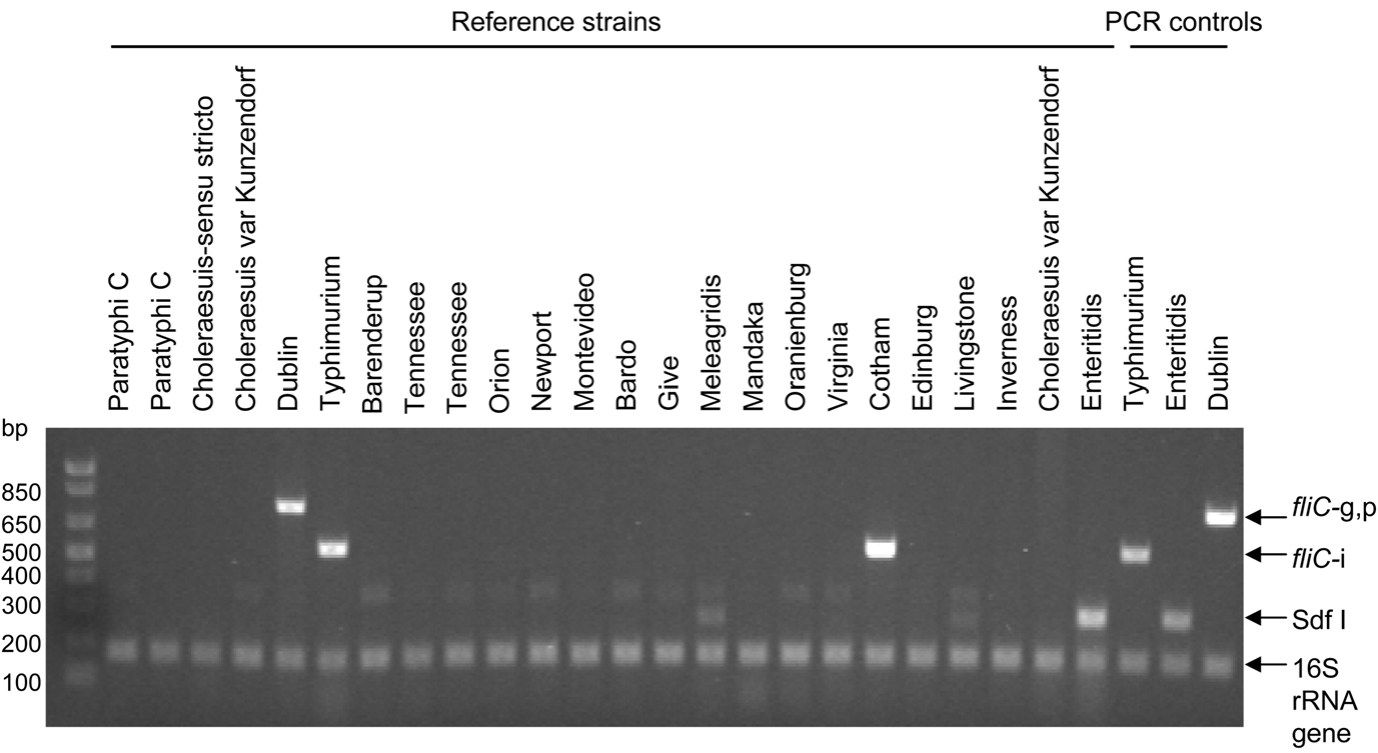
Blinded screening of 24 control strains by the *fliC*-i/*fliC*-gp/Sdf I multiplex PCR. Reference strains consisted of one representative each of *Salmonella* Typhimurium, *Salmonella* Enteritidis and *Salmonella* Dublin and a range of negative control *Salmonella* strains of various serovars. PCR controls consisted of *Salmonella* Typhimurium 81.23500, *Salmonella* Enteritidis CVD SE, *Salmonella* Dublin 06-0707.

To preliminarily assess the specificity of the multiplex PCR assay, we tested 24 control *Salmonella* strains consisting of a range of serovars (previously described in [18]) in a blinded fashion (Figure 2). The multiplex PCR correctly identified *Salmonella* Typhimurium and *Salmonella* Cotham as H:i, *Salmonella* Dublin as H:g,p and *Salmonella* Enteritidis as containing Sdf I (Figure 2). Faint products of the size of Sdf I were observed for *Salmonella* Meleagridis and *Salmonella* Livingstone. However, *Salmonella* Meleagridis is O serogroup E1 and *Salmonella* Livingstone is O serogroup C1, so when also tested by our previously described O serogrouping PCR [18], these serovars would not be mistaken as *Salmonella* Enteritidis. The same is true for *Salmonella* Cotham, which although it possesses *fliC*-i, is not O serogroup B and would not be mistaken as *Salmonella* Typhimurium. Therefore, the new multiplex PCR was sensitive in terms of its ability to identify serovar Cotham as H:i and was specific, when combined with the O-serogrouping PCR, in showing that the strain was not serovar Typhimurium. We also blind-tested a sample of *Salmonella* Typhi and *Salmonella* Paratyphi A and B strains to ensure that the PCR would not detect these strains. The multiplex PCR correctly identified *fliC*-i of six *Salmonella* Typhimurium, Sdf I of four *Salmonella* Enteritidis, and *fliC*-g,p of five *Salmonella* Dublin strains but only the 16S rRNA gene was amplified from five strains each of serovars Typhi, Paratyphi A and Paratyphi B (data not shown).

### Analysis of 327 *Salmonella* seroroup B and D clinical isolates from Mali

We blind-tested 69 non-Typhi serogroup D *Salmonella* and 258 serogroup B strains that were originally obtained from the blood cultures of febrile patients at l'Hôpital Gabriel Touré in Bamako, Mali [18] with the multiplex PCR designed to identify *Salmonella* Typhimurium (I 4,[5],12:i:1,2) and variants (monophasic I 4,[5],12:i:- and non-motile (NM) I 4,[5],12:NM), *Salmonella* Enteritidis and *Salmonella* Dublin. This PCR was performed in parallel to serotyping. We correctly identified all the serogroup D isolates (37 *Salmonella* Dublin and 32 *Salmonella* Enteritidis) and all 232 *Salmonella* Typhimurium and variant strains (Table 3). If the *Salmonella* Typhimurium-like strains (i.e., I 4,[5],12:i:- and I 4,[5],12:NM) are included in the target group then the PCR is 100% sensitive and 100% specific in identifying *Salmonella* Typhimurium, *Salmonella* Enteritidis, *Salmonella* Dublin and *Salmonella* Typhimurium-like organisms. The remaining 26 serogroup B isolates were negative for the tested targets.

**Table 3:** Detection of *Salmonella* Typhimurium and variants, *Salmonella* Enteritidis and *Salmonella* Dublin by multiplex PCR among 69 non-Typhi Group D *Salmonella* and 258 Group B *Salmonella* isolated from blood cultures of febrile patients in Bamako, Mali.

O Group	Serovar	No. of isolates	Multiplex PCR		
			*fliC-*i	*fliC-*g,p	Sdf I
D	Dublin	37	0	37	0
	Enteritidis	32	0	0	32
B	Typhimurium and Typhimurium variants	232	232	0	0
	Stanleyville	26	0	0	0

### Discrimination between *Salmonella* Typhimurium and I 4,[5],12:i:-

During the course of this study, we decided to determine the prevalence of I 4,[5],12:i:- in Mali. Levy et al. [18] identified 220 *Salmonella* Typhimurium, four I 4,[5],12:i:- and eight I 4,[5],12:NM strains. However, in this previous study, Phase 2 flagella typing was not performed on all of the strains. We re-examined the 220 *Salmonella* O serogroup B, H:i isolates that had been previously been presumptively identified as *Salmonella* Typhimurium and used classical methods (i.e., sera against the Phase 2 H_1,2_ flagella) to determine that 48 isolates were in fact I 4,[5],12:i:- (bringing the total number of isolates of this serovar to 52) and one isolate was I 4,[5],12:NM (bringing the total number of isolates of this serovar to nine). The remaining 171 strains were confirmed as *Salmonella* Typhimurium.

**Figure 3:**
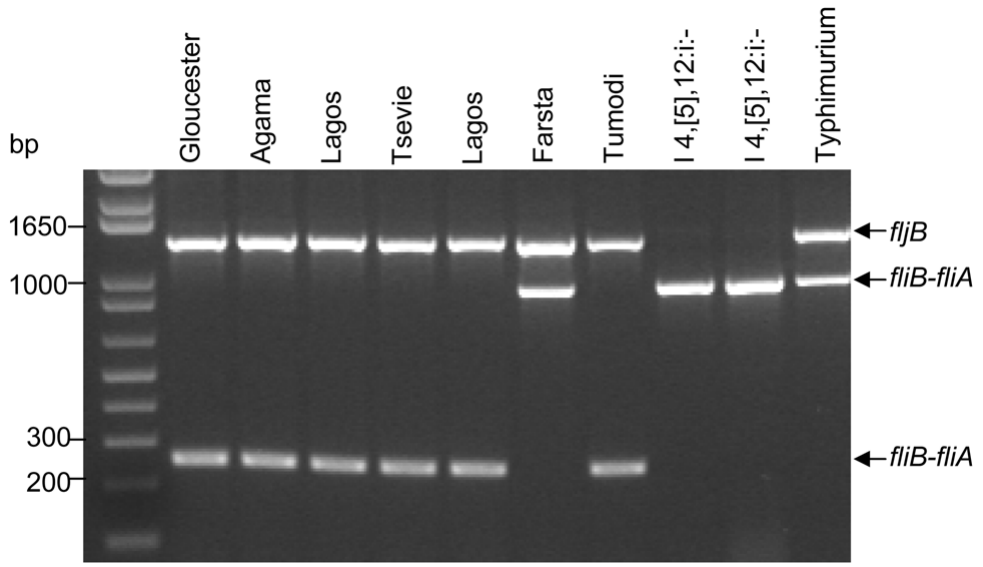
Differentiation of diphasic *Salmonella* Typhimurium and monophasic *Salmonella* Typhimurium from other H:i serovars. The PCR to discriminate between *Salmonella* Typhimurium and I 4,[5],12:i:- strains was validated using nine reference *Salmonella* O group B, H:i isolates of various serovars.

We have combined previously described primers in a PCR to discriminate between *Salmonella* Typhimurium and I 4,[5],12:i:-. Primers FFLIB and RFLIA amplify the *fliB-fliA* intergenic region of the flagellin gene cluster [24]. *Salmonella* Typhimurium strains possess an IS200 fragment in this region [25]. Burnens et al. [25] showed that 21 of 23 isolates of *Salmonella* Typhimurium and none of 85 isolates of 37 other *Salmonella* serovars contained IS200 in this region. Primers FFLIB and RFLIA have been reported to amplify a 1-kb product from *Salmonella* Typhimurium and I 4,[5],12:i:- strains and a 250-bp product from all other serovars [24]. However, when validating these primers, we found that a 1-kb fragment was amplified from *Salmonella* Farsta (not tested by Echeita et al. [24]) suggesting that this serovar also possesses IS200 in the *fliB-fliA* intergenic region (Figure 3).

Primers Sense-59 and Antisense-83 amplify the *fljB* allele [26]. Primer Sense-59 binds at position 258 and primer Antisense-83 binds at position +100 of the 5′-3′ consensus *fljB_1,2_* sequence. These primers amplify a 1389-bp product from strains that possess a Phase 2 flagellar antigen and no product from strains that lack a Phase 2 flagellar antigen such as I 4,[5],12:i:-. As shown in Figure 3, the PCR was able to discriminate between *Salmonella* Typhimurium and I 4,[5],12:i:- strains and other serogroup B, H:i serovars except *Salmonella* Farsta.

We tested all the *Salmonella* Typhimurium, I 4,[5],12:i:- and I 4,[5],12:NM strains identified in Mali and found that 170 of 171 *Salmonella* Typhimurium strains were correctly identified (i.e., possessed a 1-kb *fliB-fliA* intergenic region product and *fljB_1,2_*), and 51 of 52 I 4,[5],12:i:- strains were correctly identified (i.e., possessed a 1-kb *fliB-fliA* intergenic region product and lacked *fljB_1,2_*) (Table 4). The nine I 4,[5],12:NM strains produced mixed results in that all nine strains produced a 1-kb *fliB-fliA* intergenic region product but three strains possessed *fljB_1,2_*.

**Table 4:** Detection of *Salmonella* Typhimurium, I 4,[5],12:i:- and I 4,[5],12:NM by PCR among 232 Group B *Salmonella* that possess *fliC-i*.

Serovar	No. of isolates	Multiplex PCR		
		*fliB-fliA* intergenic region - 250 bp	*fliB-fliA* intergenic region - 1 kb	*fljB*
Typhimurium	171	0	171	170
I 4,[5],12:i:-	52	0	52	1
I 4,[5],12:NM^a^	9	0	9	3

a NM, non-motile.

### Detection of *Salmonella* Stanleyville

**Figure 4:**
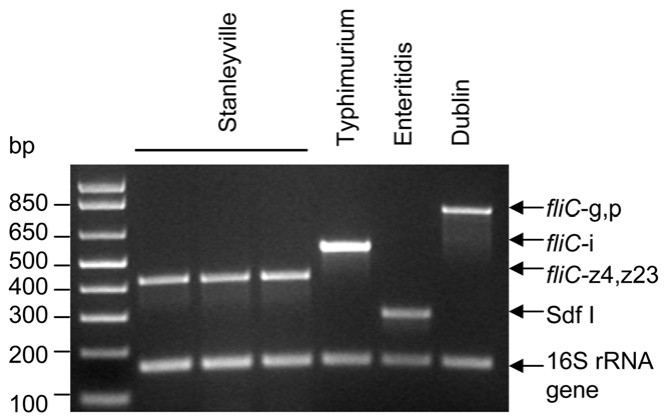
Detection of *Salmonella* Stanleyville. Amplification of *fliC* from 3 *Salmonella* Stanleyville strains from Mali and *Salmonella* Typhimurium 81.23500, *Salmonella* Enteritidis CVD SE and *Salmonella* Dublin 06-0707 following the addition of Hz4,z23 primers to the *fliC*-i/*fliC*-gp/Sdf I multiplex PCR mix.

Since *Salmonella* Stanleyville was found to be fairly common among the Mali NTS isolates, we added primers to detect *fliC-*z4,z23 of *Salmonella* Stanleyville to the multiplex PCR containing primers H-for, Hi, sdfF, sdfR, 16SF and DG74. The primers were first tested on *Salmonella* Stanleyville by themselves and produced a 427-bp amplicon. The *fliC-*z4,z23 primers were then added to the multiplex primer mix and PCR was performed (using the previously optimized conditions) on all 26 *Salmonella* Stanleyville strains, and a sample of 10 *Salmonella* Typhimurium, 10 *Salmonella* Dublin and 11 *Salmonella* Enteritidis strains. Correct amplicons were observed for all the strains tested. Figure 4 shows amplicons from a sample of three *Salmonella* Stanleyville strains and the control *Salmonella* Typhimurium, *Salmonella* Enteritidis and *Salmonella* Dublin strains.

## Discussion

We have combined published primers and new primers in a multiplex PCR that, following the application of a previously described O serogrouping multiplex PCR [18], can identify *Salmonella* Typhimurium (and variants), *Salmonella* Enteritidis, *Salmonella* Dublin and *Salmonella* Stanleyville. Detection of *Salmonella* Typhimurium, *Salmonella* Dublin and *Salmonella* Stanleyville is based on amplification of the respective *fliC* alleles. We were unable to design primers to detect *fliC-*g,m of *Salmonella* Enteritidis due to the high nucleotide identity between *fliC-*g,m and *fliC-*g,p (of *Salmonella* Dublin). We therefore used primers to detect “*Salmonella* difference fragment I” (Sdf I), a segment of *Salmonella* Enteritidis DNA that was reported to be absent from 73 non-Enteritidis *Salmonella enterica* isolates comprising 34 different serovars as determined by PCR [27]. We confirmed the utility of Sdf I, with the exception of serovars Meleagridis and Livingstone. We found that *Salmonella* Livingstone yielded a weak PCR product using the same Sdf I primers that were previously reported [27]. The disparity could be due to a difference in the amplification method (different polymerases and cycling conditions were used).

From the epidemiologic and public health perspective, being able to detect strains that are genetically similar to *Salmonella* Typhimurium yet that constitute distinct serovars (i.e., I 4,[5],12:i:- and I 4,[5],12:NM) is important (e.g., for outbreak investigations). In the USA and Europe such strains are increasingly being reported [28]–[30]. In Spain, I 4,[5],12:i:- was the fourth most commonly isolated *Salmonella* serovar from humans from 1998–1999 [29] and several studies suggest that this monophasic serovar is a variant of *Salmonella* Typhimurium [24], [31]–[33]. The PCR that we have described can generally discriminate the diphasic *Salmonella* Typhimurium serovar (I 4,[5],12:i:1,2) from monophasic (I 4,[5],12:i:-) variants. Only one *Salmonella* Typhimurium was misidentified as I 4,[5],12:i:- and vice versa. It is possible that our PCR will not be able to detect some serologically monophasic I 4,[5],12:i- strains as lack of Phase 2 flagellar antigen expression can be due to a variety of mechanisms ranging from point mutations to partial or complete deletions in *fljB_1,2_* and adjacent genes. Additionally, if there is a deletion in the first 250 bp of *fljB_1,2_*, the primers we have chosen will not identify the strain as I 4,[5],12:i-. Furthermore, our PCR scheme cannot differentiate between *Salmonella* Typhimurium and *Salmonella* Farsta. However, in practical terms, this is unlikely to pose a problem as *Salmonella* Farsta is extremely rare.

One small set of strains where our PCR gives differing results from traditional serological methods are *Salmonella* Typhimurium-like non-motile variants (I 4,[5],12:NM). Notably, all nine Malian strains identified by serotyping methods as I 4,[5],12:NM were found to possess the *fliC-i* allele and three of the strains also possessed the *fljB_1,2_* gene. Two quite distinct explanations can account for these observations. One is that in some strains lack of motility is not due to loss of flagellar genes but rather to other factors (e.g., regulation) that keep expression turned off. Alternatively, it may be that our genetic identification of these strains is correct and that the failure to detect flagella phenotypically is merely a consequence of not knowing how to grow the bacteria under conditions optimal for expression of those flagella. We assume that the I 4,[5],12:NM strains from Mali are *Salmonella* Typhimurium variants as they possess *fliC*-i and IS200 in the *fliB-fliA* intergenic region. It is also possible, albeit unlikely, that they could be the very rarely isolated *Salmonella* Farsta.

Soyer et al. [34] have reported that there are at least two common clones of I 4,[5],12:i:- with different genomic deletions (an ‘American’ deletion genotype and a ‘Spanish’ deletion genotype). Both I 4,[5],12:i:- clones completely lack *fljB* and *fljA*. Preliminary analysis of the deletion using a variety of primers that amplify different sections of the *fljB_1,2_* gene indicates that the I 4,[5],12:i:- strains from Mali appear to possess the 3′ end of *fljB* and the entire *fljA* ORF. At least 250 bp of *fljB* (including the Sense-59 binding site) has been deleted at the 5′ end (data not shown). This suggests that these strains are genetically different from both the Spanish and American I 4,[5],12:i:- isolates. We are sequencing the deletion in several Malian I 4,[5],12:i- strains to determine the exact deletion. It will be interesting to see whether I 4,[5],12:i:- strains from other African countries are genetically similar to the Malian strains.

Several other DNA-based *Salmonella* typing methods have been described [35]–[41]. However, some of these do not identify the breadth of enteric fever and NTS serovars of our multistep, multiplex PCR or fail to include an internal positive control. An O serogroup-specific Bio-Plex assay to detect serogroups B, C1, C2, D, E and O13 and serovar Paratyphi A [42] and a DNA sequence-based approach to serotyping have also been described [43]. However, these methods require greater financial and technical resources over those required for our method. Our PCRs are novel because they use as few primers as possible to identify the most common non-typhoidal *Salmonella* serovars isolated from blood and other invasive sites in sub-Saharan Africa, including *Salmonella* Typhimurium (and several variants), *Salmonella* Enteritidis, *Salmonella* Dublin and *Salmonella* Stanleyville. Since the late 1980s, the majority (85 to 95%) of NTS associated with invasive disease in sub-Saharan Africa belong to these serovars [9], [11]–[13],[15],[16],[18],[19]. Therefore, we do not believe that there is a need for multiplex PCRs that detect more serovars unless the epidemiologic picture changes. We have tried to keep the PCRs as simple as possible so that they can be performed easily and the results interpreted correctly in laboratories in Africa that may be new to PCR. If a large outbreak or otherwise frequent isolation occurred of a serovar not presently recognized or contained within our multiplex, this serovar would not be identifiable using our PCR and would have to be identified in a reference laboratory using antisera or by molecular serotyping.

**Figure 5:**
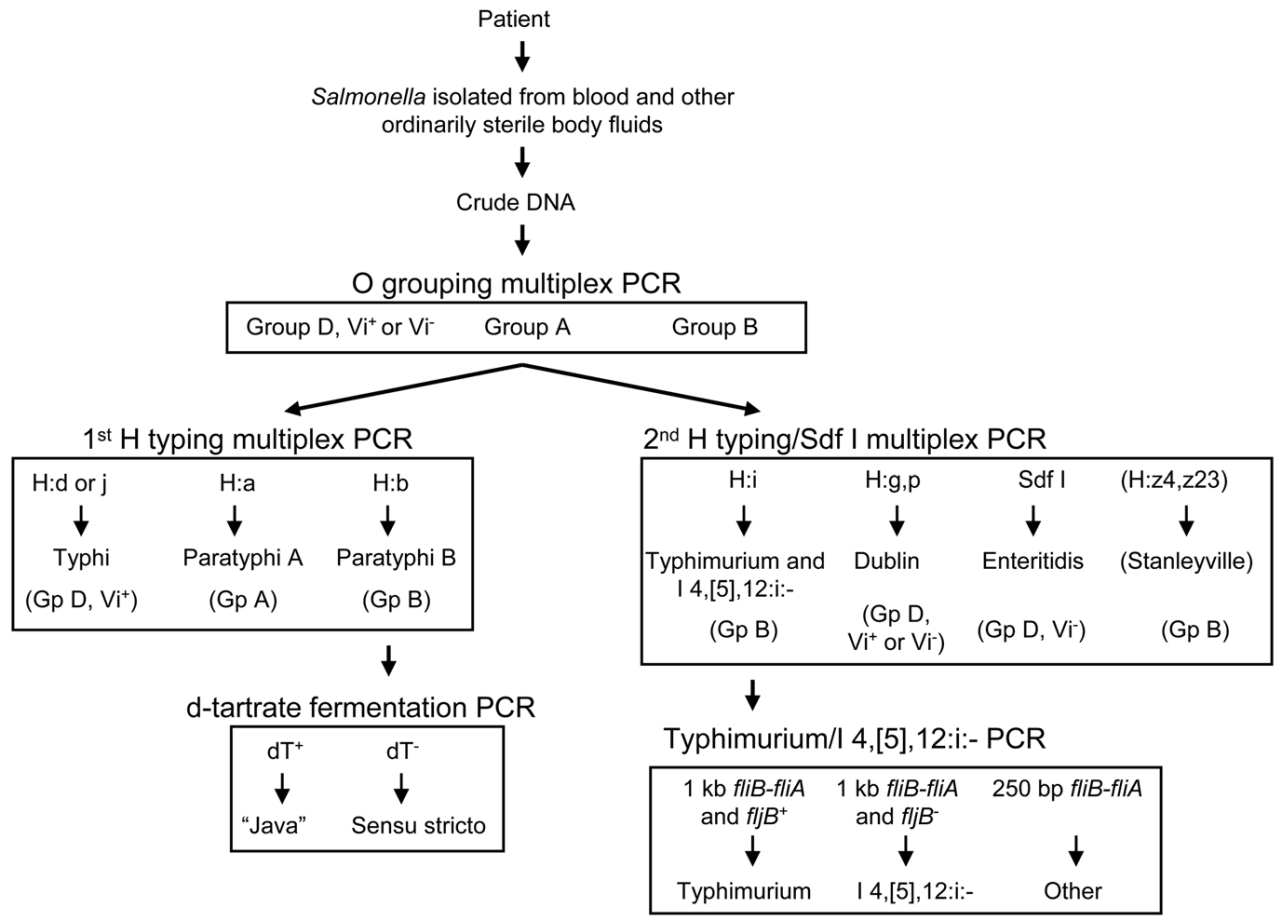
The steps used to identify typhoidal and non-typhoidal serovars of *Salmonella* isolated from blood.

We are currently evaluating various PCR reagents that are stable at room-temperature and can be readily obtained by laboratories in Africa. Depending on the prevalence of certain serovars in a given country, either typhoidal or non-typhoidal *Salmonella* (or both) can be identified using our primer sets (Table 5 and Figure 5). For example, one may wish to test all *Salmonella* isolates in the O serogrouping PCR, then screen serogroup A, B and D Vi^+^ strains using the first H typing multiplex PCR to identify *Salmonella* Typhi, *Salmonella* Paratyphi A and *Salmonella* Paratyphi B. The d-tartrate fermentation PCR can be performed to differentiate *Salmonella* Paratyphi B *sensu stricto* strains from *Salmonella* Paratyphi B Java. Any serogroup B isolates not identified by the 1^st^ H typing PCR can be tested along with non-Typhi O serogroup D strains in the second H typing/Sdf I multiplex PCR to identify serovars Typhimurium (and related strains), Dublin (which can be Vi^+^ or Vi^-^ [44]), Enteritidis and Stanleyville. The O serogroup B H:i strains can be tested using the Typhimurium/I 4,[5],12:i:- PCR to identify *Salmonella* Typhimurium and I 4,[5],12:i:-. It should be stressed that the O serogrouping PCR described by Levy et al. [18] needs to be performed in conjunction with the PCRs described here to ensure that *Salmonella* Enteritidis and *Salmonella* Typhimurium are identified correctly and not mistaken as *Salmonella* Meleagridis and *Salmonella* Livingstone; and *Salmonella* Cotham, respectively.

**Table 5:** *Salmonella* serovars identified by the PCRs described in this report and in Levy et al. [18].

	O Group	Vi	Phase 1 H flagella	Sdf I	*fliB-fliA* intergenic region	*fljB*	d-tartrate fermentation	Serovar
**Typhoidal serovars**	A	–	a	–	250 bp	+	ND^a^	Paratyphi A
	B	–	b	–	250 bp	+	–	Paratyphi B *sensu stricto*
	D	+	d or j	–	250 bp	–	ND	Typhi
**Non-typhoidal serovars**	B	–	b	ND	ND	ND	+	Paratyphi B Java
	B	–	i	–	1 kb	+	ND	Typhimurium and Farsta
	B	–	i	–	1 kb	–	ND	I 4,[5],12:i:-
	B	–	z4,z23	–	250 bp	–	ND	Stanleyville
	D	+/–	g,p	–	250 bp	–	ND	Dublin
	D	–		+	250 bp	–	ND	Enteritidis

a ND, not determined.

The surveillance experience in Mali is the first to show that *Salmonella* Dublin and *Salmonella* Stanleyville can constitute important serovars associated with invasive non-typhoidal *Salmonella* disease, along with *Salmonella* Typhimurium (and variants) and *Salmonella* Enteritidis. Previously, *Salmonella* Dublin and *Salmonella* Stanleyville were recovered only occasionally from blood cultures of patients in Africa [12],[13],[45],[46]. We thought it useful to be able to detect these serovars by PCR in future surveillance studies in Africa.

In conclusion, we have described a series of PCRs based on O serogrouping and H typing that can identify the causative agents of enteric fever (*Salmonella* Typhi and *Salmonella* Paratyphi A and *Salmonella* Paratyphi B), the three most commonly isolated serovars that cause invasive disease in young children in sub-Sahara African (*Salmonella* Typhimurium [and Typhimurium-like], *Salmonella* Enteritidis and *Salmonella* Dublin) and *Salmonella* Stanleyville, an invasive pathogen that may be of regional importance in West Africa.

## Supporting Information

Alternative Language Abstract S1 French translation of the abstract by SOS.
(0.02 MB DOC)
