# Supplementary material for: Identification by PCR of Non-typhoidal Salmonella enterica Serovars Associated with Invasive Infections among Febrile Patients in Mali
Source: PLoS Negl Trop Dis. 2010 Mar 9;4(3):e621. doi: 10.1371/journal.pntd.0000621 (PMC2834738; doi:10.1371/journal.pntd.0000621)
Supplement: Alternative Language Abstract S1 — French translation of the abstract by SOS. (0.02 MB DOC) [file pntd.0000621.s001.doc]

The French translation of the Abstract was prepared by co-author, Dr. Samba O. Sow.

**Résumé**

***Introduction:*** *Salmonella* non-typhoïdique (NTS) est en train d’émerger comme une cause éminente de maladie invasive (bactériémie ou infections focalisées comme la méningite) chez les nourrissons et les jeunes enfants en Afrique sub-Saharienne. Les données, dont celles du Mali, montrent, de façon importante, que trois serovars : *Salmonella* *enterica* Typhimurium, *Salmonella* Enteritidis et *Salmonella* Dublin constituent la majorité de NTS isolés chez ces patients.

***Méthodes:*** Nous avons étendu des séries de Polymerase Chain Reactions (PCRs) précédemment développées sur la base du sérogroupage O et du sérotypage H pour identifier *Salmonella* Typhimurium et ses variants (surtout I 4,[5],12:i:-), *Salmonella* Enteritidis et *Salmonella* Dublin. Nous avons aussi fait des primers pour détecter *Salmonella* Stanleyville, un serovar trouvé en Afrique de l’Ouest. Un autre PCR a été utilisé pour différencier *Salmonella* Typhimurium diphasique et *Salmonella* Typhimurium monophasique d’autres sérogroupes O, B, H:i serovars. Nous avons utilisé ces PCRs pour tester en aveugle des souches de 327 *Salmonella* sérogroupe B et D qui étaient obtenues d’hémocultures d’enfants fébriles à Bamako, Mali.

***Principaux résultats:*** Nous avons démontré lorsqu’il est utilisé en conjonction avec notre PRC sérogroupe O, précédemment décrit, nos PCRs sont à 100% sensibles et spécifiques dans l’identification de *Salmonella* Typhimurium et ses variants, *Salmonella* Enteritidis, *Salmonella* Dublin et *Salmonella* Stanleyville. Quand nous avons tenté de différencier les souches de 171 *Salmonella* Typhimurium (I 4,[5],12:i:1,2) des souches de 52 *Salmonella* Typhimurium (I 4,[5],12:i:-) monophasiques, une souche qui était initialement identifiée par sérologie comme chacun de ces serovars a été déterminée par PCR comme étant le contraire.

***Conclusion:*** Nous avons décrit une méthode simple, pourtant efficace de PCR pour faire la surveillance de l’incidence de la maladie invasive causée par NTS dans les pays en développement.
